# Parental report–based assessment of sleep problems and mental health in an unreferred cohort of children with a fragile X premutation

**DOI:** 10.3389/fnmol.2026.1820313

**Published:** 2026-07-15

**Authors:** Nicole D. Tortora, Jessica Ezzell Hunter, Anne Glicksman, Tiffany McGuire, Shira Russell-Giller, Amanda Kenepp, Shantal Taveras, Sonia Seehra, Rachel Goldman, Veronica J. Hinton, Lisa Shubeck, Emily Graves Allen, Tatyana Adayev

**Affiliations:** 1 Department of Human Genetics, NYS Institute for Basic Research in Developmental Disabilities (IBR), Staten Island, NY, United States; 2 Genomics, Ethics, and Translational Research Program, RTI International, Research Triangle Park, NC, United States; 3 Queens College, City University of New York, Flushing, NY, United States; 4 The Graduate Center, City University of New York, New York, NY, United States; 5 Department of Human Genetics, Emory University School of Medicine, Atlanta, GA, United States

**Keywords:** anxiety, children, depression, *FMR1*, fragile X premutation, mental health, sleep

## Abstract

**Introduction:**

Individuals with a fragile X premutation (PM) are at an increased risk for a variety of physical and mental health conditions, including sleep problems. Sleep problems in children in the general population have been shown to adversely affect development, cognition, behavior, and mood. Because information on the impact of sleep problems in children with a PM is limited, this study aimed to explore the extent of sleep problems in an unreferred cohort of children with a PM and with no PM (NP) and the relationship between sleep and mental health.

**Methods:**

Using the parent-completed Child Behavior Checklist, sleep and mental health were assessed across two distinct study samples. First, a cross-sectional preschool cohort (*n* = 324; ages 3–5 years) was used to identify early associations and CGG-moderated effects. Second, a longitudinal cohort (*n* = 128) was assessed at Time 1 (3–7 years) and Time 2 (8–13 years) to examine cross-sectional and longitudinal associations of sleep and mental health outcomes.

**Results:**

Females with a PM exhibited significantly more sleep problems than females with NP at ages 3–5 years. In the preschool cohort, CGG repeat size moderated the relationship between sleep and mental health in a sex- and symptom-specific manner. Longitudinal trajectories differed by PM status: for children with NP, early sleep problems appear stable and predict later sleep outcomes. In contrast, for children with a PM, later sleep problems appear emergent and were associated with concurrent mental health symptoms independent of earlier sleep difficulties.

**Discussion:**

The results demonstrate that females with a PM are more likely than females with NP to experience sleep problems at young ages, and the relationship between sleep and mental health in children with a PM is moderated by CGG repeat size. Children with a PM appear to follow a unique developmental pathway characterized by longitudinal trajectories that diverge from those of children with NP, suggesting different underlying mechanisms.

## 1 Introduction

Fragile X syndrome (FXS) is the most common inherited form of intellectual disabilities and is the result of a CGG trinucleotide repeat expansion in the 5′ untranslated region of the fragile X messenger ribonucleoprotein 1 (*FMR1*) gene to more than 200 CGG repeats (Rousseau et al., 1995; Crawford et al., 2001). A fragile X premutation (PM), characterized by 55 to 200 CGG repeats and the risk of expansion to a full mutation on transgenerational transmission, affects approximately 1 in 291 females and 1 in 855 males in the general population (Hunter et al., 2014). Beyond two well-established conditions affecting individuals with a PM, Fragile X-Associated Primary Ovarian Insufficiency and Fragile X-Associated Tremor/Ataxia Syndrome, individuals with a PM are also at increased risk for a variety of physical and mental health conditions often referred to as Fragile X Premutation Associated Conditions (FXPAC) (Hagerman and Hagerman, 2004; Coffey et al., 2008; Allen et al., 2020; Johnson et al., 2020). Mental health conditions are among the most common of these problems in adults with a PM, with a high incidence of anxiety and depression (Hessl et al., 2005; Roberts et al., 2009; Lachiewicz et al., 2010; Bourgeois et al., 2011; Kenna et al., 2013; Gossett et al., 2016; Hagerman et al., 2018). Emerging evidence suggests that children with a PM may also experience elevated levels of anxiety and depression similar to adults with a PM (Bailey et al., 2008; Cordeiro et al., 2015). In addition to these mental health conditions, individuals with a PM often experience significant sleep problems (Roberts et al., 2009; Chonchaiya et al., 2010; Allen et al., 2020).

Sleep problems are prevalent among children, affecting approximately 25% in the general population (Owens, 2008), and have been shown to adversely affect development, cognition, behavior, and mood (Goodlin-Jones et al., 2009; Chénier-Leduc et al., 2019; Schlieber and Han, 2021). Research has shown that preschool- and school-aged children who experience sleep problems, such as refusal to sleep alone, bedtime resistance, and sleeping less than others, have higher rates of anxiety and depression (Johnson et al., 2000; Gregory and O’Connor, 2002; Gregory et al., 2008; Alfano et al., 2009; Whalen et al., 2017; Muller et al., 2023) as well as a greater risk for suicidal thoughts and behavior (Hoyniak et al., 2023). Conversely, emotional and behavioral problems have been reported to increase the incidence of sleep problems, indicating a bidirectional relationship (Iwadare et al., 2015; Wang et al., 2016; Chénier-Leduc et al., 2019; Liu et al., 2021). Moreover, persistent sleep problems in childhood can lead to an increased likelihood of mental health outcomes in adulthood (Gregory et al., 2005).

A high prevalence of sleep problems has also been reported in neurodevelopmental disorders (NDDs), including Rett Syndrome, Angelman Syndrome, Prader-Willi Syndrome (Veatch et al., 2021), Autism Spectrum Disorder (Earl et al., 2021), and FXS (Budimirovic et al., 2022). Although the frequency of sleep problems in FXS is lower than in some other NDDs, it has still been found to affect ~23–46% of children with FXS (Budimirovic et al., 2022). Previous studies have demonstrated a strong association between sleep problems in NDDs and various mental health conditions such as anxiety and depression (Earl et al., 2021; Budimirovic et al., 2022), consistent with findings in the general population. Furthermore, caregivers and families of children with NDDs often experience elevated rates of sleep and mental health problems, underscoring the profound effect on the entire family (Veatch et al., 2021; Gálvez-Ortega et al., 2024).

Despite extensive research on the relationship between sleep and mental health problems in the general population and NDDs, little is known about this connection in children with a fragile X PM. Given the heightened risk of sleep and mental health problems in individuals with a PM, it is crucial to investigate this interplay, particularly during childhood, when intervention could have the most significant impact. In this study, we assessed the degree of sleep problems in an unreferred cohort of children with a fragile X PM and children with no PM (NP) (5 to 44 CGG repeats) and explored the association between sleep problems and mental health. By examining this association, we aimed to determine if having a PM confers a heightened vulnerability to sleep and mental health disorders in children.

## 2 Methods

### 2.1 Participants

Participants were part of a larger NIH-funded study, *Association of the Fragile X Premutation with Cognitive and Behavioral Skills in Children* (R01HD102429), with an overarching goal of characterizing neurodevelopmental outcomes in children with a PM. This study examines the development, cognition, and behavior of children with and without a fragile X PM who either had their *FMR1* status determined prenatally or were their siblings. The prenatal *FMR1* analysis was performed at the IBR Specialty Clinical Laboratories (Staten Island, NY) during prenatal workup due to familial *FMR1* expansion or a family history of FXS and included families across the United States. For these participants, genotypes were clinically determined using CGG triplet-primed PCR and Southern blot analysis. Parents were informed of their child’s PM status as part of this diagnostic process. Alternatively, PM status was verified via a clinical report provided by the parent. All eligible families were then invited to participate in the study allowing for recruitment with minimal ascertainment bias. Participants (*n* = 453) in this larger study were used to identify three distinct cohorts: (1) *Cross-sectional study group* (*n* = 325): includes all children who only had one assessment completed by their parents when they were 3–7 years old and were not part of the longitudinal study group. (2) *Longitudinal study group* (*n* = 128): includes all children who had two assessments completed by their parents, the first when they were 3–7 years old (Time 1), and the second when they were 8–13 years old (Time 2). Thus, there was no overlap between the cross-sectional and longitudinal study groups. (3) *Preschool cohort* (*n* = 324): includes the combination of children aged 3–5 years drawn from both the cross-sectional and longitudinal study groups whose parents completed the CBCL preschool version, which includes the Sleep Problems scale. This targeted combination was implemented to maximize statistical power and was utilized exclusively for these early childhood cross-sectional analyses (i.e., CGG repeat size and status-based comparisons), thereby avoiding duplication of observation across analyses. Their descriptive characteristics are presented separately in Tables 1, 2, reflecting their distinct and differing utilization across various analyses.

**Table 1:** Descriptive characteristics of participants in the cross-sectional and longitudinal study groups.

Descriptive category	PREMUTATION (PM)		NO PREMUTATION (NP)	
	Males	Females	Males	Females
Cross-sectional study group	*n* = 85	*n* = 59	*n* = 92	*n* = 89
Mean age at Time 1 (years)^a^	4.82	5.02	5.28	4.79
	3.02–7.95 (SD = 1.55)	3.04–7.93 (SD = 1.55)	3.03–7.96 (SD = 1.53)	3.04–7.73 (SD = 1.24)
Mean age at Time 2 (years)	N/A	N/A	N/A	N/A
PM sizes, CGGn (min-max)	65 (55–140)	62 (55–120)	N/A	N/A
Ascertainment				
Prenatal	90.6%	83.1%	87%	85.4%
Sibling	9.4%	16.9%	13%	14.6%
Race and ethnicity				
White, Non-Hispanic	81.2%	83.1%	82.6%	69.7%
Black, Non-Hispanic	1.2%	1.7%	0%	3.4%
Asian, Non-Hispanic	5.9%	3.4%	2.2%	9.0%
Hispanic (of any race)	7.1%	10.1%	8.7%	9.0%
Other	4.6%	1.7%	6.5%	7.9%
Not reported	0%	0%	0%	1.0%
Maternal education				
High school/GED	0%	1.7%	2.2%	2.2%
Trade or technical school	1.2%	3.4%	0%	1.1%
Some college	0%	5.1%	1.1%	2.2%
2- or 4-year college degree	25.9%	27.1%	31.5%	30.4%
Graduate or professional degree	69.4%	57.6%	57.6%	60.7%
Not reported	3.5%	5.1%	7.6%	3.4%
Longitudinal study group	*n* = 35	*n* = 27	*n* = 35	*n* = 31
Mean age at Time 1 (years)	5.24	4.99	5.4	4.98
	3.07–7.31 (SD = 1.12)	3.01–7.45 (SD = 1.26)	3.03–7.75 (SD = 1.5)	3.08–7.52 (SD = 1.33)
Mean age at Time 2 (years)	10.5	10.5	10.20	10.20
	8.00–13.7 (SD = 1.77)	8.06–13.20 (SD = 1.63)	8.38–13.6 (SD = 1.49)	8.05–13.0 (SD = 1.72)
PM sizes, CGGn (min-max)	63 (55–175)	63 (55–119)	N/A	N/A
Ascertainment				
Prenatal	91.4%	85.2%	74.3%	90.3%
Sibling	8.6%	14.8%	25.7%	9.7%
Race and ethnicity*				
White, Non-Hispanic	85.7%	81.5%	80%	77.4%
Black, Non-Hispanic	0%	0%	0%	3.2%
Asian, Non-Hispanic	0%	0%	11.4%	12.9%
Hispanic (of any race)	0%	14.8%	0%	0%
Other	14.3%	3.7%	8.6%	6.5%

CGGn: Number of CGG repeats; SD: Standard Deviation.

a Significant difference between males with PM and NP (*p* = 0.05), as determined by Welch Two Sample *t*-test.

*Significant difference between PM and NP groups (*p* < 0.05), as determined by Fisher’s Exact test.

**Table 2:** Descriptive characteristics for the preschool cohort.

Descriptive category	PREMUTATION (PM)		NO PREMUTATION (NP)	
	Males	Females	Males	Females
	*n* = 86	*n* = 65	*n* = 78	*n* = 95
Mean age	4.22	4.38	4.33	4.35
	3.02–5.90 (SD = 0.86)	3.01–5.99 (SD = 0.95)	3.03–5.98 (SD = 0.94)	3.04–5.97 (SD = 0.83)
PM sizes, CGGn (min–max)	64 (55–175)	62 (55–120)	N/A	N/A
Ascertainment				
Prenatal	90.7%	84.6%	93.6%	88.4%
Sibling	9.3%	15.4%	6.4%	11.6%
Race and ethnicity				
White, Non-Hispanic	81.4%	81.6%	79.5%	69.5%
Black, Non-Hispanic	1.2%	0%	0%	3.2%
Asian, Non-Hispanic	3.5%	1.5%	5.1%	9.5%
Hispanic (of any race)	2.3%	10.7%	2.6%	4.2%
Other	11.6%	6.2%	12.8%	13.6%
Maternal education				
High school/GED	0%	1.5%	0%	2.1%
Trade or technical school	1.2%	3.1%	0%	1.1%
Some college	0%	3.1%	0%	2.1%
2- or 4-year college degree	33.6%	33.9%	24.4%	31.6%
Graduate or professional degree	64.0%	56.9%	67.9%	58.9%
Not reported	1.2%	1.5%	7.7%	4.2%

CGGn, Number of CGG repeats; SD, Standard Deviation.

The longitudinal study cohort included children whose parents provided data at two separate timepoints. Assessment timepoints were determined by the parent study protocol, which recruited children aged 3–13 years for parent-completed surveys and initiated follow-up direct assessment once participants reached age 8. To maximize data collection, parents were invited to complete surveys a second time even if the child was not involved in direct assessment due to parental choice, provided at least 1 year had elapsed since their initial assessment. The average interval between the Time 1 and Time 2 assessments was 5.2 years (range: 1.1 to 8.5 years). To handle this interval range, we included age at test as a covariate in every model to control for the developmental stage at each assessment.

Information about medication use was only collected at Time 1, not at Time 2. At Time 1, fewer than 2 % of children in either cohort were taking stimulants or sleep aids, thus the impact of medication on initial outcomes was likely minimal, though the lack of longitudinal medication data is a limitation.

This study was approved by the Institutional Review Board of record (IRB protocol #7328), and the parent/guardian of all children in the study gave their informed consent prior to their inclusion in the study.

### 2.2 Measures

All outcomes were captured through parent-reported completion of the age-appropriate version (preschool version for ages 1.5–5 years and school-age version for ages 6–18 years) of the Achenbach System of Empirically Based Assessment (ASEBA) Child Behavior Checklist (CBCL) (Achenbach et al., 1987; Achenbach, 1991) at Time 1 and Time 2 via the ASEBA web platform. On all items, parents were asked to rate items as 0 (not true), 1 (somewhat or sometimes true), or 2 (very true or often true) based on what best describes their child then or in the previous 6 months.

#### 2.2.1 Sleep problems

Sleep problems were coded in two ways: (1) A dichotomous variable to indicate the presence or absence of sleep problems, using a T-score of greater than or equal to 60. While the standard ASEBA diagnostic cutoff for the Sleep Problems scale considers T-scores of ≥ 65 to fall within the borderline clinical and clinical range, our selection of a ≥ 60 threshold provides a more sensitive indicator. This threshold identifies children whose parents endorsed symptoms greater than one standard deviation above the mean, representing more symptoms than expected for 84% of the normative population. This approach allows for the capture of both clinically significant and at-risk symptoms, thereby enabling the identification of more subtle differences between groups. (2) An ordinal variable for sleep score to assess sleep problems across both CBCL preschool and school-age versions that was determined based on parent responses to CBCL sleep-related items. Specifically, this score was derived from the following items on the preschool version: does not want to sleep alone, has trouble getting to sleep, nightmares, resists going to bed at night, sleeps less than most kids during the day and/or night, talks or cries out in sleep, wakes up often at night; and the school-age version: nightmares, overtired without good reason, sleeps less than most kids, sleeps more than most kids during the day and/or at night, talks or walks in sleep, trouble sleeping. This method, adapted from a previously published study (Stoléru et al., 1997), allowed for consistent measurement across both CBCL forms. Although this method was not formally validated, an internal comparison was conducted between the determined sleep score and standardized CBCL Sleep Problems T-scores in the preschool version. The results showed a robust linear correlation (*r* = 0.61, *p* < 0.001), supporting the use of this sleep score as a cross-form measure for sleep difficulties (see Supplementary Table 1). Briefly, to determine the sleep score: if the parent rated any item a 2 (very true or often true), the child was given a sleep score of 2. The child was also given a sleep score of 2 if the parent rated any three sleep items a 1 (somewhat or sometimes true). A sleep score of 2 is considered the highest level of sleep problems experienced by children. If the parent rated one or two sleep items a 1, the child was given a sleep score of 1. If the parent rated all sleep items a 0 (not true), the child was given a sleep score of 0. Children with a sleep score of 0 were considered to have no sleep problems.

#### 2.2.2 Mental health

Either T-scores or total raw scores were used depending on the analyses and cohort being examined. For cross-sectional comparisons within the preschool cohort, age-standardized T-scores were utilized for the Depressive Problems and Anxiety Problems subscales from the CBCL. Total raw scores for the Depressive Problems and Anxiety Problems subscales were used to assess the child’s mental health at Time 1 and Time 2 for the longitudinal study cohort and Time 1 for the cross-sectional study cohort. The total raw score is calculated by summing the ratings for all items within that subscale (10 items for each subscale for the preschool version of the CBCL, 13 items for the Depressive Problems subscale, and nine items for the Anxiety Problems subscale of the school-age version). In all cases, higher scores indicate a greater symptom burden.

### 2.3 Statistical analysis

Statistical analysis was conducted using R version 4.4.2 (R Core Team, 2024). An alpha level of *p* = 0.05 was established as the baseline threshold for statistical significance. To control for multiple comparisons across our primary models, corrections were applied using the Benjamini-Hochberg False Discovery Rate (FDR) procedure, with statistical significance set at *p*FDR < 0.05. Stratification by sex was pre-specified based on well-documented sex-differential gene expression and phenotypic variability in the *FMR1* PM. Due to the limited diversity of the sample, race was coded as a binary variable, with participants classified as White and Non-White.

### 2.4 Cohort-specific analysis

#### 2.4.1 Cross-sectional analysis of the preschool cohort (ages 3–5)

##### 2.4.1.1 Sleep problems and having a PM

To assess the extent of sleep problems in children aged 3–5 with a PM, the preschool cohort, which included all participants with Sleep Problems scale data, was utilized. This dataset included 324 children: 151 children with a PM [86 males, 65 females] and 173 with NP [78 males, 95 females] (Table 2). Chi-square analyses were used to assess sleep problems (coded as a dichotomous variable) between children with and without a PM. To account for the differential gene expression between the sexes due to the X-linked nature of the *FMR1* gene, these analyses were performed for all children as a group as well as for males and females separately. In addition, for children with a PM, the model was tested using CGG repeat size as a continuous variable to determine if there is an association between repeat size and sleep problems. To account for the possibility of a non-linear relationship, both linear and non-linear (quadratic) analyses were conducted for the total sample and stratified by sex.

##### 2.4.1.2 CGG repeat size and sleep-related mental health outcomes

To investigate if CGG repeat size influences mental health outcomes, the interaction between CGG repeat size and sleep problems was examined as a predictor of mental health in the preschool cohort. Using linear regression models, sleep and mental health outcomes were assessed using standardized T-scores. Prior to conducting the analyses, the data were screened for outliers using Cook’s Distance; one participant (a male with 99 CGG repeats) was identified as a significant outlier (*D* = 77.9), exerting disproportionate leverage on the quadratic interaction terms. Therefore, he was excluded to ensure the robustness of the regression models. All models were adjusted for the child’s age at test. To account for potential sex-specific phenotypic expressions, analyses were conducted separately by sex for each mental health outcome (Depression and Anxiety). Furthermore, both linear and non-linear interactions were tested to determine if the interaction between sleep and repeat size followed a linear or curvilinear relationship.

#### 2.4.2 Longitudinal analysis (ages 3–13)

**Figure 1:**
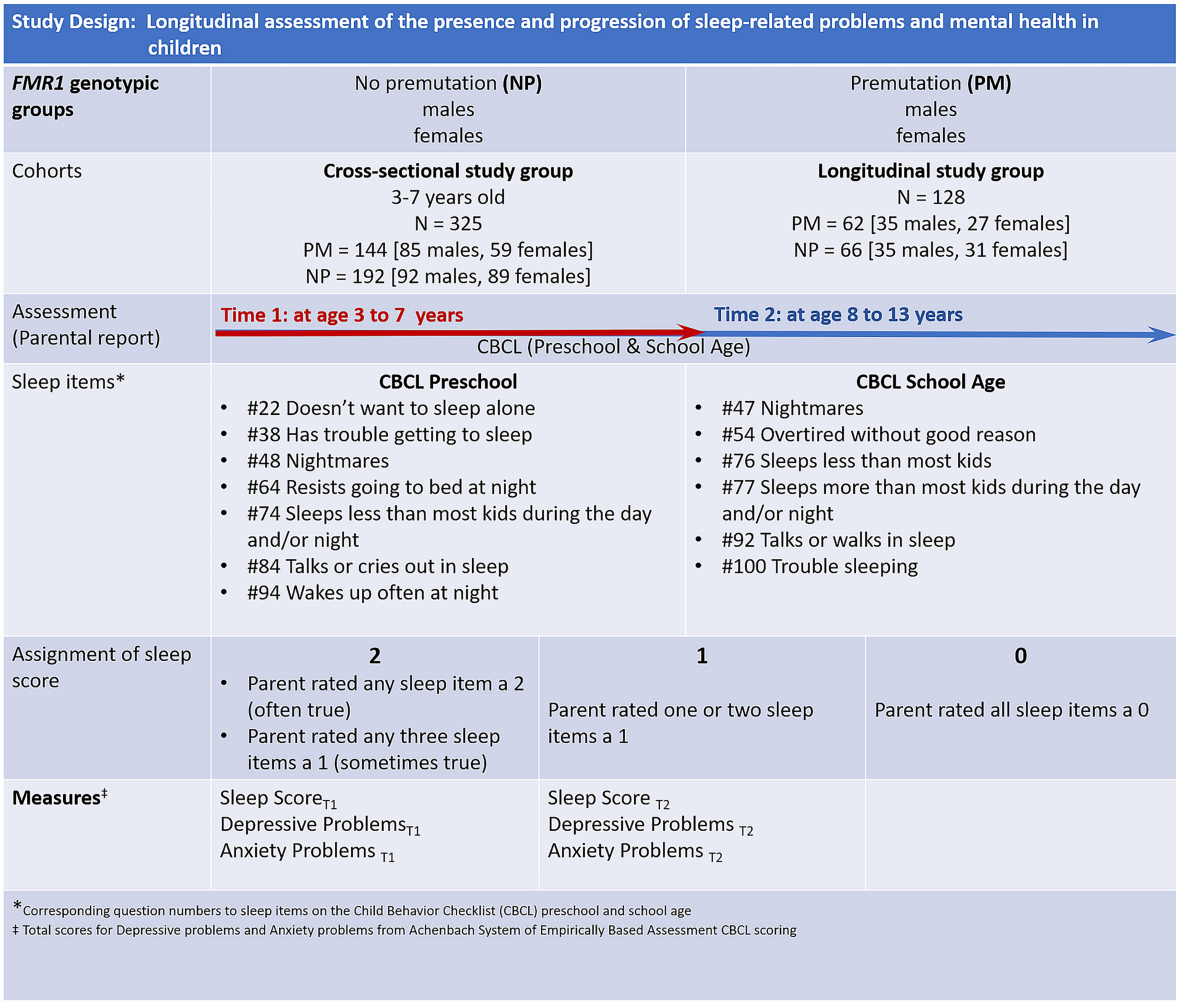
Study design overview for the longitudinal assessment of children with and without a PM.

For the longitudinal analysis, children were assessed with and without a PM to determine the extent of sleep-related problems and the association of sleep and mental health problems by following the study design outlined in Figure 1.

All of the following analyses were limited to the longitudinal study cohort unless otherwise noted. Analyses were performed for all children, as well as stratified by sex and by having a PM to identify group-specific developmental trajectories that are often masked in global models. Separate analyses were performed for depression and anxiety. For models run on the group as a whole, adjustments were made for child’s age at test, fragile X status, sex, and race; for analyses of individual groups, only age at test and race were included as covariates.

While the relationship between CGG repeat size and mental health was a primary focus in the larger preschool cohort, it was not included as a predictor in the longitudinal analyses. Given the reduced sample size of the longitudinal cohort, the data lacked sufficient statistical power to reliably model the effects of the wide range of CGG repeat sizes as a covariate alongside multiple covariates and interaction terms. Therefore, longitudinal analyses were restricted to examining the progression of sleep problems and mental health symptoms independently of CGG repeat size.

##### 2.4.2.1 Sleep problems as a predictor of mental health problems

To assess the association between sleep and mental health at each timepoint, analysis of variance (ANOVA) was used to compare mean mental health scores across sleep scores (coded as a 3-level ordinal variable) at the two timepoints. Linear regression analysis was run using sleep problems as a predictor of mental health problems at each timepoint. Specifically, sleep problems were used at Time 1 as a predictor of mental health problems at Time 1, and sleep problems at Time 2 as a predictor of mental health problems at Time 2. To account for potential confounding effects, models were adjusted for the child’s age at test, fragile X status, sex, and race.

##### 2.4.2.2 Sleep problems at Time 2

A linear regression model was used to examine the association between sleep problems at Time 1 and sleep problems at Time 2, controlling for age at test, time difference between assessments, fragile X status, sex, and race. Subsequent linear regression analysis was used to examine the relationship between mental health and sleep at Time 2. Hence, both the longitudinal association (Time 1 mental health as a predictor of Time 2 sleep) and the concurrent association (Time 2 mental health and Time 2 sleep) were assessed, while controlling for the same covariates.

##### 2.4.2.3 Sensitivity analysis

Linear regression analysis was used to compare the cross-sectional study group with the longitudinal study group. This analysis was conducted to assess the robustness and generalizability of the associations between sleep problems at Time 1 and mental health symptoms at Time 1, confirming that the findings from the smaller longitudinal cohort were consistent with those of the larger, cross-sectional sample.

Additionally, a sensitivity analysis was conducted for the cross-sectional preschool cohort using the standard ASEBA clinical cutoff of a T-score ≥ 65 to evaluate the impact of a stricter clinical threshold on the differences in sleep problems between females with and without a PM.

## 3 Results

### 3.1 Cross-sectional analysis of the preschool cohort (ages 3–5)

#### 3.1.1 Sleep problems and having a PM

**Figure 2:**
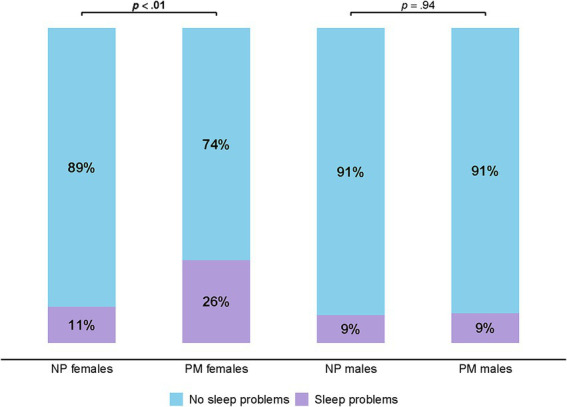
Percentage of children with and without a PM with sleep problems by sex. A cutoff score of T = 60 on the sleep problems scale in the Child Behavior Checklist preschool version was used to define the presence of sleep problems. Chi-square analysis was conducted to determine the significance of observed differences. PM, premutation; NP, no premutation.

In analyses of children aged 3–5 years using a T-score of 60 or greater to indicate the presence of sleep problems, no significant difference was found in the presence of sleep problems between children with or without a PM (*χ*^2^(1) = 3.24, *p* = 0.07). In analyses stratified by sex, females with a PM had significantly more sleep problems than females with NP (*χ*^2^(1) = 6.72, *p* < 0.01), with an odds ratio of 3.01 (95% CI: 1.28–7.1). No significant difference was seen for males (*χ*^2^(1) = 0.01, *p = 0*.94). Figure 2 represents the percentage of parent-reported sleep problems in male and female children with and without a PM.

There was no significant association between CGG repeat size and sleep problems in the combined group of children with a PM (*β* = 0.008, *p* = 0.82). When examined by sex, CGG repeat size was not significantly associated with sleep problems in either females (*β* = −0.004, *p* = 0.95), or males (*β* = 0.015, *p* = 0.65). Testing for non-linear relationships revealed no significant associations between repeat size and sleep problems for the total group or for either sex (all *p*’s > 0.05).

Welch two sample *t*-tests and Fisher’s exact analyses revealed no significant differences between the PM and NP groups across any of the descriptive characteristics, nor were there any significant sex differences within the cohort.

#### 3.1.2 CGG repeat size and sleep-related mental health outcomes

**Figure 3:**
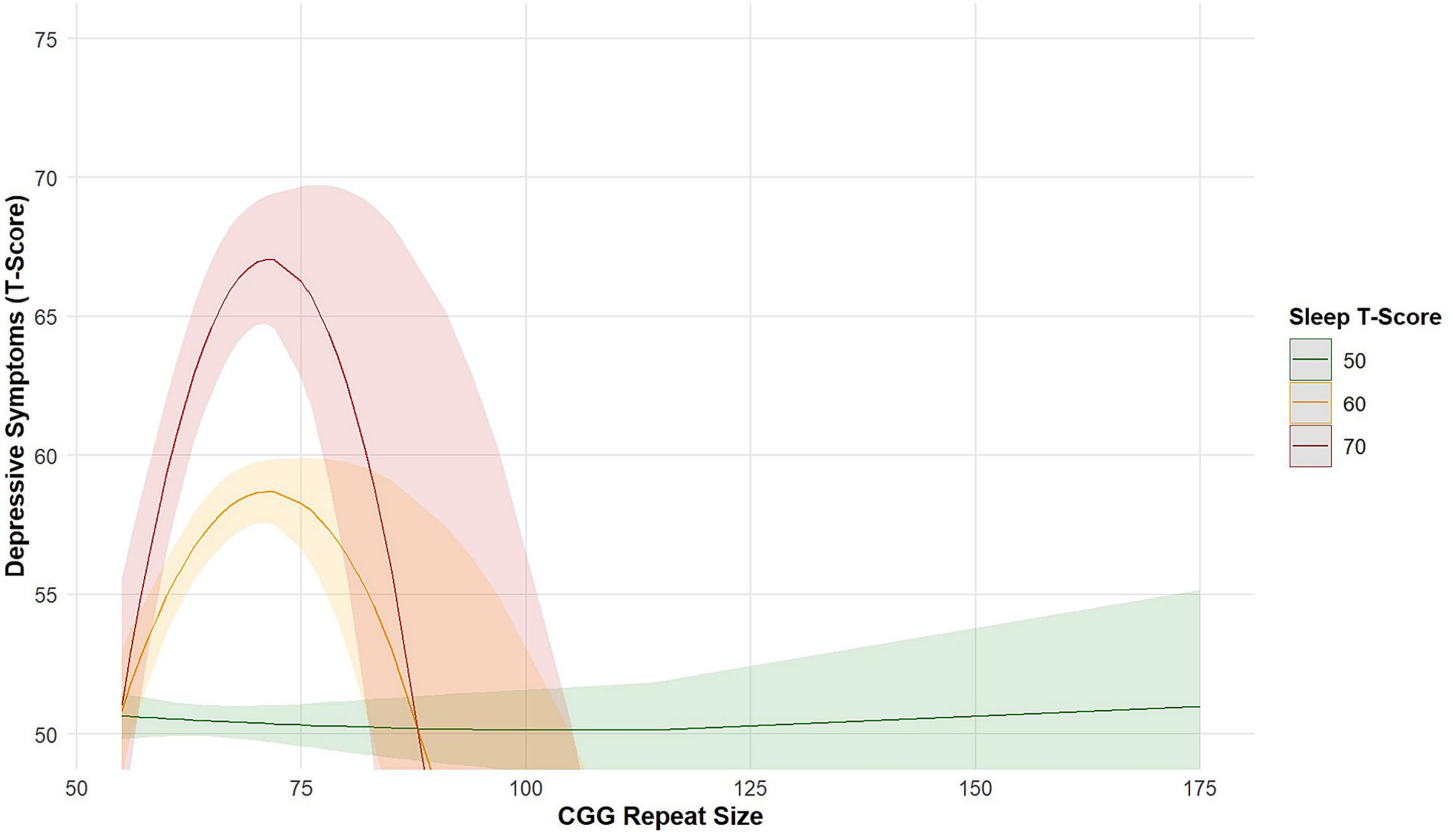
Predicted depressive symptoms as a function of the curvilinear interaction between CGG repeat size and sleep problems in males with a PM. Lines represent predicted T-scores at three levels of sleep disruption: 50 (average), 60 (at-risk), and 70 (clinically significant). The model illustrates a non-linear compounding effect (*F*(6, 78) = 31.75, *p* < 0.001, adj. *R*^2^ = 0.687), with the greatest vulnerability occurring in the mid-range CGG repeat size.

For males with a PM (*n* = 85, following the removal of one outlier), we evaluated the compounding effect of sleep problems and CGG expansion on both depressive and anxiety symptoms by testing linear and non-linear (quadratic) interaction models. For depression, the interaction was first tested using a standard linear framework, with results indicating a significant linear interaction (*β* = 0.033, *p*FDR < 0.001). To test for non-linear effects, subsequent analysis adding a second-order polynomial for CGG repeat size, also yielded a significant curvilinear interaction (*β* = −0.003, *p*FDR = 0.002). A formal comparison confirmed that the curvilinear model provided a significantly superior fit over the linear model, (*F*(2, 78) = 7.27, *p* = 0.001). This selection was further supported by a lower Akaike information criterion (AIC) for the curvilinear model (377.01) compared to the linear model (387.54), and an increase in variance explained (adj. *R*^2^ = 0.687 vs. 0.638). As illustrated in Figure 3, predicted values demonstrate that the compounding influence of sleep problems on depression reaches a peak intensity in the mid-expansion range.

A similar approach was utilized to analyze anxiety symptoms in males with a PM. In contrast to the findings for depressive symptoms, there was no significant linear interaction of sleep and CGG repeat size (*β* = 0.013, *p*FDR = 0.36), or non-linear interaction (*β* = −0.003, *p*FDR = 0.14) on anxiety.

**Figure 4:**
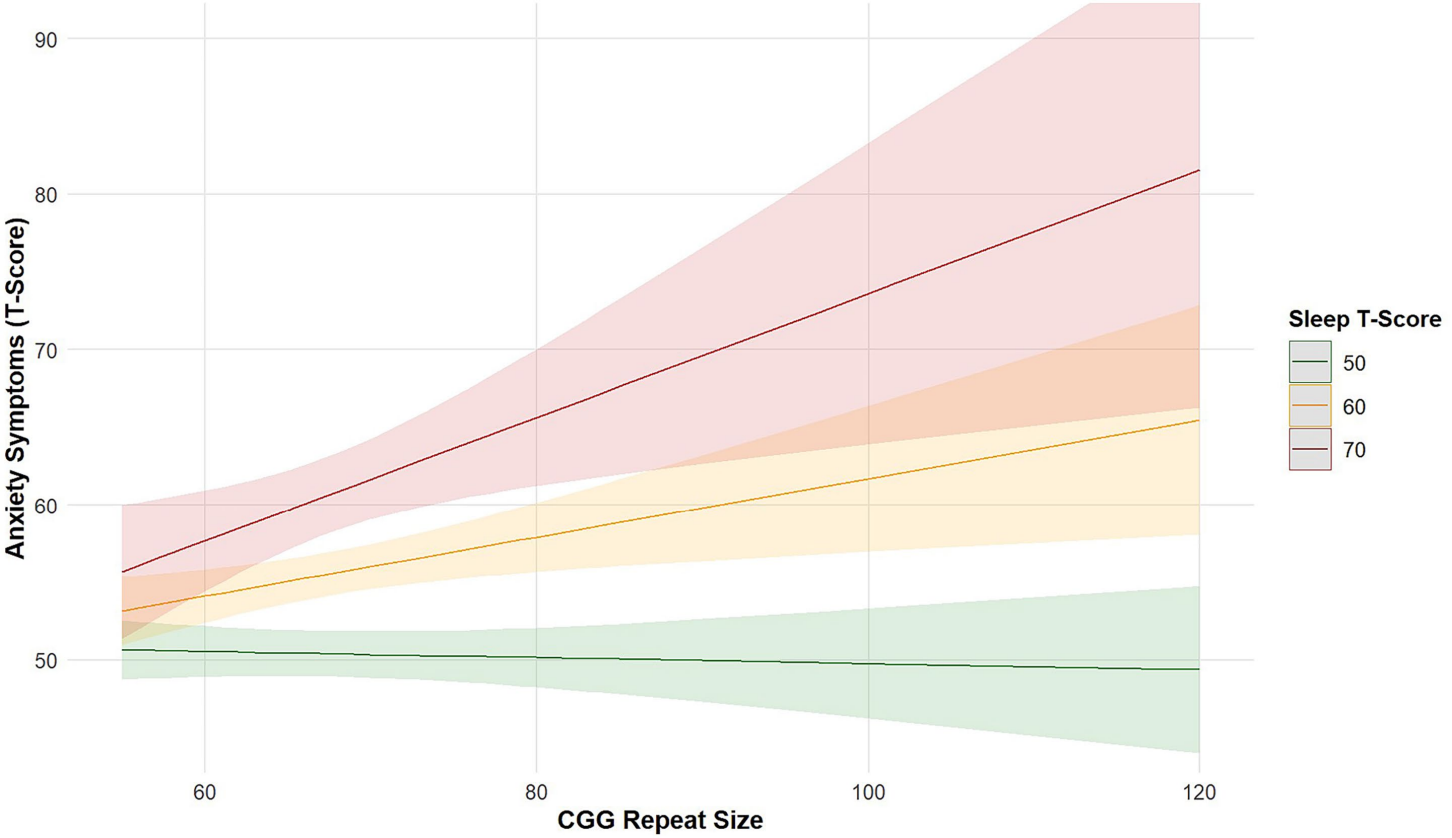
Predicted anxiety symptoms in females with a PM as a function of the linear interaction between CGG repeat size and sleep problems. Lines represent predicted T-scores at three levels of sleep disruption: 50 (average), 60 (at-risk), and 70 (clinically significant). This linear compounding effect (*F*(4, 60) = 13.71, *p* < 0.001, adj. *R*^2^ = 0.443,) demonstrates that the impact of sleep disruption on anxiety symptoms escalates steadily as CGG repeat size increases.

For females with a PM (*n* = 65), the same comparative modeling approach was used to examine the relationship between sleep problems and CGG repeats. While sleep problems remained a robust independent predictor of depression in females with a PM, the interaction between sleep and CGG repeat size did not reach significance in either the linear (*β* = −0.009, *p*FDR = 0.30) or the curvilinear models (*β* = 0.001, *p*FDR = 0.30). Interestingly, the results for anxiety symptoms in females with a PM favored a linear rather than a curvilinear framework. In the linear model, a significant interaction between sleep problems and CGG repeat size (*β* = 0.021, *p*FDR = 0.036) was found, indicating that as CGG repeats increase, the association between sleep and anxiety becomes progressively stronger (Figure 4). However, when testing for non-linear effects, the quadratic interaction terms were not significant (*β* = −0.001, *p*FDR = 0.30), and the model fit did not significantly improve.

### 3.2 Longitudinal analysis (ages 3–13)

#### 3.2.1 Sleep problems as a predictor of mental health problems

**Figure 5:**
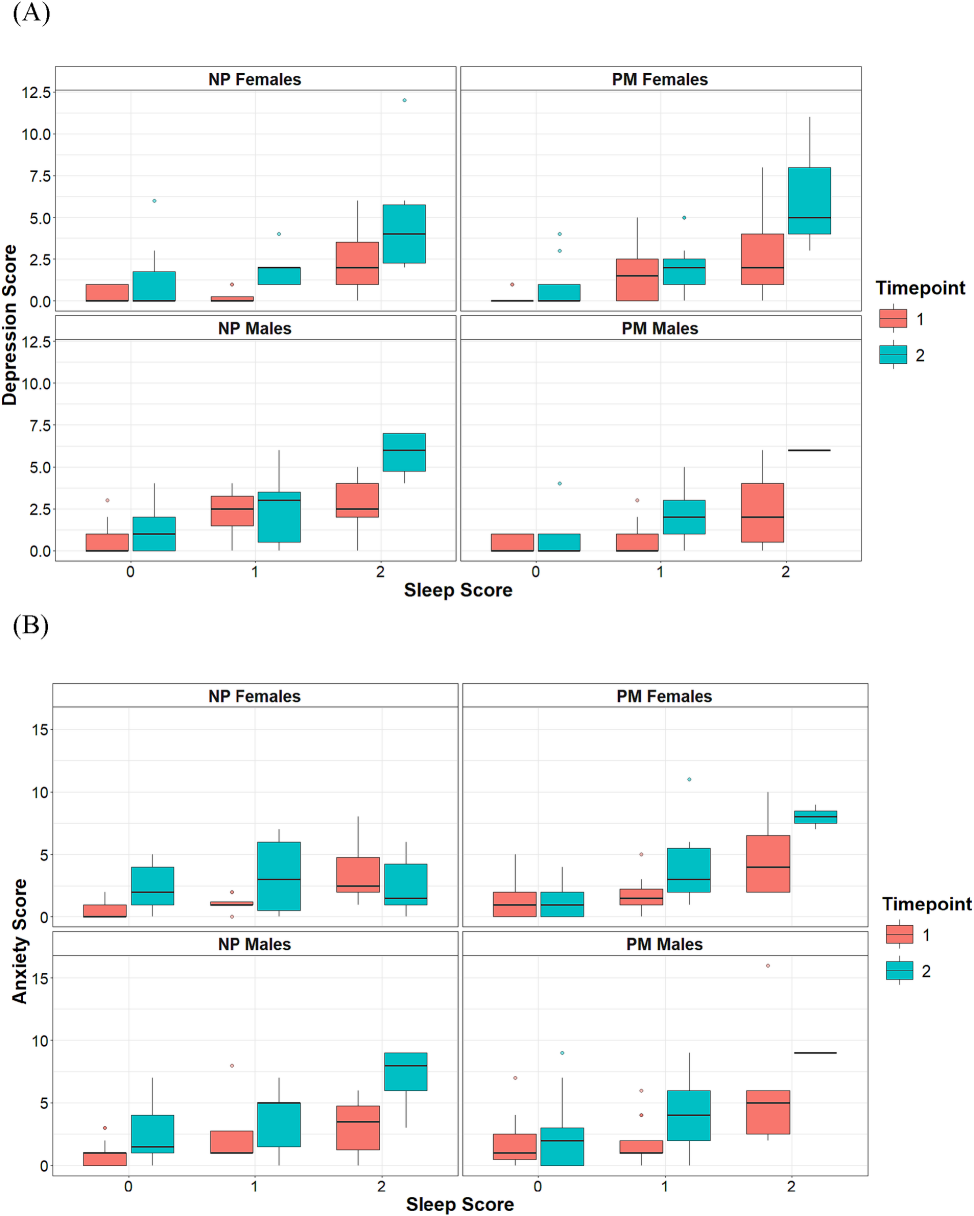
Mean depression **(A)** and anxiety **(B)** scores for individual groups across sleep scores at timepoints 1 and 2. Sleep score: 0, no sleep problems; 1, some sleep problems; 2, highest level of sleep problems. NP, no premutation; PM, premutation.

In unadjusted analyses, higher sleep scores were generally associated with increased severity of depression and anxiety symptoms at both timepoints (Table 3 and Figure 5). ANOVA confirmed these associations were statistically significant for depression across all groups. For anxiety, significant associations were also observed for most groups at both timepoints, except for females with NP at Time 2 (*p* = 0.60). While the overall trend was positive, several of the analyses revealed a threshold effect, with depression and anxiety symptoms rising sharply between sleep scores of 1 and 2.

**Table 3:** Mean mental health scores by sleep score and timepoint.

Mental health category	Sleep score at each timepoint	All participants		PM females		NP females		PM Males		NP males	
		*n* = 128		*n* = 27		*n* = 31		*n* = 35		*n* = 35	
		T1_Mean Score	T2_Mean Score	T1_Mean Score	T2_Mean Score	T1_Mean Score	T2_Mean Score	T1_Mean Score	T2_Mean Score	T1_Mean Score	T2_Mean Score
Depression	0	0.4	0.92	0.17	0.77	0.31	1	0.36	0.58	0.62	1.21
	1	1	2.17	1.75	2.18	0.25	1.86	0.71	2.2	2.25	2.43
	2	2.59	5.57	2.86	6.33	2.3	5	2.43	6	2.8	5.75
ANOVA											
F-statistic		51.5	86.57	11.63	18.45	12.41	14.91	9.83	29.53	24.4	30.26
DF		1	1	1	1	1	1	1	1	1	1
*p*		**< 0.001**	**< 0.001**	**0.002**	**< 0.001**	**0.001**	**< 0.001**	**0.004**	**< 0.001**	**< 0.001**	**< 0.001**
Anxiety	0	1.07	2.03	1.33	1.15	0.38	2.22	1.91	2.16	0.9	2.25
	1	1.78	3.83	1.88	4.09	1.12	3.29	1.82	4	2.75	3.57
	2	4.03	5.43	4.71	8	3.3	2.5	5.71	9	3.1	7
ANOVA											
*F*-statistic		39.32	31.7	10.44	32.77	24.56	0.29	6.1	8.66	11.71	14.7
DF		1	1	1	1	1	1	1	1	1	1
*p*		**< 0.001**	**< 0.001**	**0.003**	**< 0.001**	**< 0.001**	**0.**60	**0.019**	**0.006**	**0.002**	**< 0.001**

T1 = Time 1 (3–7 years old); T2 = Time 2 (8–13 years old).

PM, Premutation; NP, No Premutation; ANOVA, Analysis of Variance; DF, Degrees of freedom.

Significant *p*-values (*p*) are bold (*p* < 0.05).

Similar results were seen when using linear regression analysis. After adjusting for potential confounding effects, sleep problems at Time 1 were significantly associated with both depression and anxiety at Time 1, across all groups. At Time 2, sleep problems were significantly associated with depression and anxiety at Time 2 for all groups except females with NP, where the association with anxiety was not significant (*p*FDR = 0.603) (Table 4). While statistically significant demographic differences were observed for race and maternal education between PM and NP groups, only race was included as a covariate in the final regression models, as maternal education did not meaningfully confound the primary associations (coefficient change < 10%). Full model results are detailed in Supplementary Table 2.

**Table 4:** Linear regression analysis of sleep and mental health at Time 1 and Time 2.

Participant group	T1 sleep score → T1 depressive problems			T1 sleep score → T1 anxiety problems			T2 sleep score → T2 depressive problems			T2 sleep score → T2 anxiety problems		
	*β*	SE	*p*FDR	*β*	SE	*p*FDR	*β*	SE	*p*FDR	*β*	SE	*p*FDR
All participants *n* = 128	1.08	0.15	**< 0.001**	1.46	0.23	**< 0.001**	2.01	0.22	**< 0.001**	1.75	0.32	**< 0.001**
PM females *n* = 27	1.32	0.42	**0.005**	1.79	0.53	**0.004**	2.33	0.57	**< 0.001**	3.29	0.60	**< 0.001**
NP females *n* = 31	1.06	0.29	**0.002**	1.37	0.33	**< 0.001**	1.86	0.48	**0.001**	0.27	0.52	0.603
PM males *n* = 35	0.79	0.33	**0.025**	1.84	0.76	**0.023**	2.08	0.37	**< 0.001**	2.30	0.83	**0.010**
NP males *n* = 35	1.11	0.23	**< 0.001**	1.08	0.33	**0.004**	1.87	0.37	**< 0.001**	2.06	0.57	**0.002**

T1 = Time 1 (3–7 years old); T2 = Time 2 (8–13 years old).

*β*: The change in mental health for a 1 unit change in sleep score.

*p*FDR: Benjamini-Hochberg False Discovery Rate-adjusted *p*-value, PM, Premutation; NP, No Premutation, SE, Standard Error.

Significant *p*-values are bold (*p*FDR < 0.05).

#### 3.2.2 Sleep problems at Time 2

Given that sleep problems were significantly associated with mental health symptoms at both Time 1 and Time 2, further longitudinal analyses were run to identify the specific factors associated with sleep problems at Time 2. Sleep problems at Time 1 showed a significant longitudinal association with sleep problems at Time 2 for the group as a whole (*β* = 0.32, *p*FDR < 0.001); however, in analysis of the individual groups, this association was limited to females with NP (*β* = 0.52, *p*FDR = 0.009) and males with NP (*β* = 0.45, *p*FDR < 0.001). No significant association was seen for females with a PM (*β* = 0.25, *p*FDR = 0.21) or males with a PM (*β* = −0.09, *p*FDR = 0.55).

The longitudinal relationship between early mental health (Time 1) and later sleep (Time 2) appeared symptom- and group-specific. Conversely, mental health at Time 2 was significantly and concurrently associated with sleep problems at Time 2 across all individual groups, except for anxiety in females with NP (*β* = 0.04, *p*FDR = *0*.666) (Table 5).

**Table 5:** Association of sleep problems at Time 2 and mental health at Time 1 and 2.

Participant group	T1 depressive problems → T2 sleep score			T2 depressive problems → T2 sleep score			T1 anxiety problems → T2 sleep score			T2 anxiety problems → T2 sleep score		
	*β* (SE)	*p*FDR		*β* (SE)	*p*FDR		*β* (SE)	*p*FDR		*β* (SE)	*p*FDR	
All participants	0.13 (0.04)	**0.002**		0.20 (0.02)	**< 0.001**	*p*	0.07 (0.03)	**0.008**	*p*	0.12 (0.02)	**< 0.001**	*p*
*n* = 128												
Adjusted *R*^2^	0.083			0.411			0.059			0.196		
*F*-statistic	2.914		*p* **= 0.01**	15.78		*p* **< 0.001**	2.325		*p =* **0.037**	6.162		*p* **< 0.001**
PM females	0.13 (0.07)	0.094		0.18 (0.04)	**0.002**		0.14 (0.05)	**0.023**		0.17 (0.03)	**< 0.001**	
*n* = 27												
Adjusted *R*^2^	0.062			0.359			0.174			0.522		
*F*-statistic	1.437		*p* **=** 0.26	4.637		*p* **= 0.007**	2.372		*p =* 0.084	8.084		*p* **< 0.001**
NP females	0.22 (0.10)	0.050		0.19 (0.05)	**0.002**		0.11 (0.09)	0.287		0.04 (0.07)	0.666	
*n* = 31												
Adjusted *R*^2^	0.051			0.262			−0.072			−0.12		
*F*-statistic	1.401		*p* **=** 0.26	3.667		*p* **= 0.017**	0.495		*p =* 0.74	0.195		*p =* 0.939
PM males	0.03 (0.08)	0.763		0.25 (0.04)	**< 0.001**		0.002 (0.03)	0.949		0.1 (0.03)	**0.009**	
*n* = 35												
Adjusted *R*^2^	−0.019			0.516			−0.023			0.217		
*F*-statistic	0.838		*p* **=** 0.51	10.06		*p* **< 0.001**	0.806		*p =* 0.531	3.355		*p* = **0.022**
NP males	0.15 (0.08)	0.075		0.24 (0.05)	**< 0.001**		0.17 (0.06)	**0.009**		0.14 (0.04)	**0.004**	
*n* = 35												
Adjusted *R*^2^	0.079			0.448			0.205			0.246		
*F*-statistic	1.729		*p* **=** 0.17	7.888		*p* **< 0.001**	3.197		*p =* **0.027**	3.766		*p* = **0.013**

*β*: The change in sleep score for 1 unit change in mental health.

*p*FDR: Benjamini-Hochberg False Discovery Rate-adjusted *p*-value, PM, Premutation; NP, No Premutation; SE, Standard error.

Significant *p*-values are bold (*p* < 0.05, *p*FDR < 0.05).

#### 3.2.3 Sensitivity analysis

A significant difference in age at test was observed between males with and without a PM in the cross-sectional cohort; however, this was accounted for in the analyses. Sensitivity analysis validated the primary findings, demonstrating a significant association between sleep problems at Time 1 and depression (*β* = 1.18, *p* < 0.001) and anxiety (*β* = 1.69, *p* < 0.001) at Time 1.

When re-running the analysis at the stricter standard clinical cutoff (T-score ≥ 65), the difference in sleep problems between females with and without a PM did not reach statistical significance, though a marginal trend was observed [Fisher’s exact test *p* = 0.070; OR = 3.17, 95% CI (0.80, 15.05)]. Only 12 females total met this higher severity threshold (8 PM vs. 4 NP). While this limited the statistical power, the direction of the group difference was consistent with the primary findings, with the sample estimate reflecting an observed increase in the odds of clinical sleep problems for females with a PM compared to females with NP.

## 4 Discussion

In an unreferred cohort of children with known *FMR1* status, we studied whether children with a fragile X PM are more likely to experience sleep problems than children with NP and determined if sleep problems are associated with mental health problems. Furthermore, within the preschool-aged cohort, we investigated whether the relationship between sleep and mental health was moderated by CGG repeat size.

Using the sensitive CBCL threshold (T-score ≥ 60), sleep problems were reported for all categories of participants; however, we found that preschool-age females with a PM exhibited a significantly higher percentage of sleep problems compared to preschool-age females with NP, whereas no difference was observed in males. When evaluating this group difference against the stricter standard clinical cutoff (T-score ≥ 65), the relationship shifted to a marginal trend. While this shift appeared to be related to a notable reduction in sample power, the sample estimate remained quite stable, with an odds ratio that closely tracked our primary finding.

Sleep problems have only been found to affect ~10% of our study’s control group (children with NP), which differs from the estimate in the general population (~25%) (Owens, 2008). This difference may be attributable to several factors. For example, our sample is not representative of the general population, as all mothers have a known *FMR1* expansion. This may introduce a unique environmental influence, as mothers with a PM may also have children with FXS and may be more attuned to different developmental and behavioral issues. This heightened awareness may lead to proactive parenting for their children with NP, including earlier intervention for behavioral problems. Additionally, there may be a reporting bias where parents perceive fewer problems in their children with NP when comparing them to their children with a PM or FXS. These factors, along with other unknown variables, may contribute to the lower prevalence of sleep problems observed in our control group.

A primary finding in our preschool cohort is that the relationship between sleep problems and mental health in children with a PM is not uniform but is instead moderated by CGG repeat size. Importantly, while CGG repeat size was not a significant independent predictor of sleep or mental health on its own, it functioned as a powerful catalyst when paired with sleep disruption. Our results align with a growing body of literature suggesting that the PM phenotype is uniquely sensitive to environmental stressors. Specifically, our finding that sleep problems and CGG repeats interact to predict mental health mirrors the framework established in studies of mothers with a PM, where the expansion size and environmental stressors create a compounding risk. For example, Seltzer et al. (2012) demonstrated that CGG repeat size significantly interacts with negative life events to predict symptoms of maternal anxiety and depression. Similarly, Hartley et al. (2012) found that the X-activation ratio (XAR) interacted with the frequency of child behavioral problems to predict physiological stress (morning cortisol) in mothers with a PM. Extending this to internal stressors, Dembo et al. (2023) showed that the interaction between CGG repeat size and poor sleep quality predicted negative health outcomes among women with a PM. While these previous studies focused on adult mothers, our findings extend this framework to the pediatric population and suggest that internal physiological disruptions, such as sleep problems, can act as potent stressors from an early age. It appears the *FMR1* expansion may not act as an independent predictor of mental health but instead serves to make individuals more susceptible to the effects of stressors, whether those stressors are external life events or internal physiological disruptions like sleep problems.

However, the specific manifestation of this susceptibility appears sex- and symptom-specific. For females with a PM, the interaction followed a direct linear pattern specifically for anxiety symptoms, where higher CGG repeats compounded the effect of sleep problems. In contrast, for males with a PM, the interaction was most robust for depressive symptoms, and was characterized by a curvilinear relationship. Specifically, the compounding influence of sleep problems reached its peak intensity in the mid-expansion range. This finding of mid-range vulnerability adds to a complex body of literature regarding the expression of the PM phenotype. These sex differences should be interpreted with caution, however, given the variation in CGG ranges within our sample. While the male cohort included expansions up to 175 repeats, the female range was narrower, spanning up to 120. It is possible that the linear pattern observed in females reflects a segment of a broader curvilinear relationship that was not fully captured due to the absence of higher CGG repeat sizes in the female group. While some studies have suggested linear relationships between CGG repeats and clinical symptoms (Hunter et al., 2008; Shelton et al., 2014), others have observed that those in the middle of the expansion range (typically ~80–100 repeats) may be at the greatest risk for specific medical, neurological, or behavioral challenges (Roberts et al., 2009; Seltzer et al., 2012; Loesch et al., 2014; Mailick et al., 2014; Klusek et al., 2018, 2019; Allen et al., 2021; Dembo et al., 2023).

In the longitudinal study group as a whole, we found a robust association between sleep and mental health outcomes, which aligns with prior research (Gregory and O’Connor, 2002; Sivertsen et al., 2015; Wang et al., 2016; Whalen et al., 2017; Moo-Estrella et al., 2022; Muller et al., 2023). Moreover, our findings indicate a strong positive association between sleep problems and the severity of depression and anxiety symptoms, suggesting that females with a PM have both increased sleep and mental health problems at Time 1 (3–7 years of age).

While these findings highlight an early vulnerability for females with a PM, our longitudinal data suggests that this relationship does not follow a stable or predictable path as they age. This is evidenced by the lack of longitudinal continuity in sleep problems for children with a PM. In contrast to both males and females with NP, for whom sleep problems at Time 1 remained significantly associated with sleep problems at Time 2, we found no such longitudinal stability for either males or females with a PM. Furthermore, the groups appear to diverge as they age; females with NP showed a dissociation between anxiety and sleep at Time 2 that was absent in females with a PM. This divergence suggests different developmental trajectories between the groups over time.

Our study adds to the growing body of literature and aligns with findings from previous studies that found increased rates of depression and anxiety in children and adults with a PM compared to controls (Bailey et al., 2008; Roberts et al., 2009; Cordeiro et al., 2015; Gossett et al., 2016). Our results suggest that individuals with more severe sleep problems at Time 2 generally experience significantly higher levels of mental health symptoms than those with similar sleep scores at Time 1 (Table 3 and Figure 5), extending prior studies that demonstrate an increase in mental health problems over time (Gregory and O’Connor, 2002; Roberts et al., 2016). Nevertheless, because these results are based on parent report, we may be underestimating the level of mental health problems these children are experiencing. Research has shown that parents may underreport the severity and frequency of internalizing symptoms, such as depression and anxiety, particularly in older children who may be more adept at concealing their difficulties (Liu et al., 2021), and therefore we may not be observing the full extent of problems at Time 2.

The persistence of sleep problems in children has been linked to persistent mental health problems through adolescence and adulthood (Gregory et al., 2005; Fichter et al., 2009; Reeve and Bell, 2023) and leads to an increased risk of suicidal thoughts and behaviors after the age of 8 years (Hoyniak et al., 2023). Our findings indicate that sleep problems at Time 2 are associated with sleep problems at Time 1 only in children with NP. In contrast, our results suggest that in males and females with a PM, the strong association between sleep problems and depression and/or anxiety symptoms at Time 2, is not predicted by earlier sleep problems (Time 1). Although the bidirectionality of sleep and mental health has been studied extensively (Jansson-Fröjmark and Lindblom, 2008; Wang et al., 2016; Kortesoja et al., 2020; Liu et al., 2021), our findings indicate the possibility of distinct longitudinal trajectories for children with and without a PM. While it is difficult to determine if mental health difficulties precede or are a consequence of sleep disturbances at an early age because they already co-occur at Time 1, it is likely the mechanisms are intertwined. Given our findings and the subtle FXS profile observed in individuals with a PM (Aziz et al., 2003; Farzin et al., 2006; Bailey et al., 2008; Maltman et al., 2021), we hypothesize that the mechanisms underlying sleep and mental health problems in children with a PM are more analogous to those observed in FXS than those in the general population.

The well-established association between increased CGG repeat size, elevated mRNA levels, and reduced FMRP levels in individuals with a PM is thought to contribute to the development of this phenotype (Tassone et al., 2000a,b; Kenneson, 2001; Allen et al., 2004; Hessl et al., 2005; Hunter et al., 2008; Kenna et al., 2013; Loesch et al., 2014). Our findings in the preschool cohort offer a window into how these genetic factors may act as a ‘first hit’ within a double-hit framework. In this model, the genetic alterations represent a latent biological vulnerability that remains subclinical on its own, explaining why CGG repeat size was not a significant independent predictor in our sample. However, when paired with the ‘second hit’ of sleep disruption, this underlying vulnerability leads to the manifestation of anxiety and depressive symptoms we observed.

Our findings, in conjunction with previous research, suggest that a potential mechanism that may be involved in the development of sleep and mental health symptoms in individuals with a PM is the disruption in one of the pathways leading to the production or metabolism of serotonin and/or melatonin, both of which have been implicated in FXS. Gould et al. (2000) found higher levels of melatonin in the fragile X group at each of six sampling times than in controls, even though they exhibit greater sleep problems, suggesting a metabolic defect or dysfunctional melatonin receptors. Hanson and Hagerman (2014) propose impaired metabolic processing of tryptophan, the precursor to both serotonin and melatonin. Trials examining the therapeutic potential of sertraline, a selective serotonin reuptake inhibitor often used to treat anxiety and depression, and melatonin in FXS have shown promising results in improving sleep, development, cognition, and social aspects (Wirojanan et al., 2009; Hess et al., 2016). These findings suggest that dysregulation of serotonin and/or melatonin pathways may contribute to the core symptoms of FXS and possibly those experienced by individuals with a PM. Additionally, other genetic or epigenetic factors may contribute to the observed sleep and mental health problems, as previous studies have identified specific polymorphisms associated with aggression and stereotyped behaviors in FXS (Hessl et al., 2008), and an association between *FMR1* intron 1 methylation levels and psychiatric symptoms (Cornish et al., 2015).

While treatment in adolescents and adults in the general population has shown promise in improving sleep quality and mental health (Blake et al., 2017, 2018; Ballesio et al., 2018), our findings emphasize the need for more proactive and consistent monitoring of individuals with a PM.

### 4.1 Limitations

First and foremost, the findings are based on parent report. More objective data, such as physiological measures and diagnostic interviews, would allow for a more comprehensive assessment of sleep problems and their impact on mental health. Secondly, this cohort is not diverse, minimizing the generalizability of the findings to other populations. Additionally, the longitudinal study sample size is small, restricting our ability to study the distribution of CGG repeat sizes as a covariate in the analyses of children with a PM. Because the PM diagnostic CGG repeat range is so wide (55–200), a larger sample is needed to identify associations between CGG repeat size and mental health symptoms. Furthermore, use of the sleep scoring method, while consistent across CBCL preschool and school-age forms, has not been externally validated. Although more recently developed CBCL-based sleep scales such as that proposed by Mansolf and Blackwell (2023) offer enhanced psychometric properties, they are limited to the school-age form and cannot be applied to preschool data. Our choice to use the determined sleep score approach was dictated by the need for a consistent sleep measure across developmental stages. Nevertheless, internal validation efforts against existing CBCL-derived T-scores in the preschool sample showed comparable patterns (Supplementary Table 1), supporting the scoring strategy used in this study. A further limitation is that medication use was only captured at Time 1, preventing its inclusion as a covariate in our longitudinal analyses. Although the number of children on medication at Time 1 was small, this may not accurately reflect medication use at Time 2. Capturing this information at multiple timepoints would strengthen future studies. Moreover, while the broader parent study protocol captures comprehensive neurodevelopmental metrics—such as cognitive scores, executive functions, and autism traits —these data are currently in the process of being collected and could not be included as covariates, representing potential unmeasured confounders in the sleep–mental health relationship. Lastly, other possible confounders such as environmental factors and parental mental health status were not evaluated. Future studies should consider these limitations when designing methods to further elucidate the complex relationship between sleep and mental health in this population. Specifically, verifying these parent-report findings against objective, direct assessment measures will be essential for proper risk assessment.

### 4.2 Strengths

The strengths of this study far outweigh its limitations and contribute to the currently sparse literature in a significant way. This research utilizes the largest unreferred sample of children with a PM ascertained through prenatal diagnosis, providing a unique opportunity to assess sleep problems and mental health in a non-clinical cohort. Notably, the identification of sleep problems and mental health symptoms relies on standardized reports rather than just anecdotal feedback, ensuring a more rigorous evaluation. Furthermore, fragile X status is confirmed through genetic testing and is not based on parent report. The longitudinal design allows us to examine how the relationship between sleep problems and mental health evolves over time and enables us to identify how these associations differ across developmental stages of a child’s life.

## 5 Conclusion

Our findings indicate that females with a PM exhibit more sleep problems than females with NP at a young age, and greater sleep disturbances are associated with increased symptoms of depression and anxiety. Of particular interest is the interaction observed in the 3–5-year-old preschool cohort, where the relationship between sleep and mental health was found to vary as a function of CGG repeat size. This suggests that the PM expansion may contribute to a heightened biological sensitivity that is more readily apparent when paired with physiological stressors like sleep disruption. Furthermore, the longitudinal associations between sleep and mental health appear to differ by group. For children with NP, development is characterized by stability and predictability, where early sleep problems reliably forecast later sleep outcomes which are closely tied to mental health outcomes. In contrast, children with a PM demonstrate a lack of longitudinal continuity, with symptoms appearing emergent and remaining primarily concurrent.

To the best of our knowledge, this is the first study to potentially identify a unique pathway for sleep and mental health problems in children with a fragile X PM, providing the foundational work for future studies.

## Data Availability

The data supporting the conclusions of this study are available from the corresponding author upon reasonable request.
