# Supplementary material for: Parental report–based assessment of sleep problems and mental health in an unreferred cohort of children with a fragile X premutation
Source: Front Mol Neurosci. 2026 Jul 15;19:1820313. doi: 10.3389/fnmol.2026.1820313 (PMC13416670; doi:10.3389/fnmol.2026.1820313)
Supplement: Supplementary file 1 [file Data_Sheet_1.PDF]

Correlative validation of CBCL preschool sleep scores and sleep problems T-scores

	Sleep & Depressive Problems			Sleep & Anxiety Problems		
	β	SE	*p*	β	SE	*p*
***All participants:* n= 324**						
Sleep score	1.13	0.10	**< .001**	1.70	0.16	**< .001**
Sleep T-score	0.20	0.01	**< .001**	0.26	0.02	**< .001**
***PM Females:* n= 65**						
Sleep score	1.49	0.29	**< .001**	1.95	0.33	**< .001**
Sleep T-score	0.22	0.02	**< .001**	0.25	0.03	**< .001**
***NP Females:* n= 95**						
Sleep score	1.19	0.20	**< .001**	1.87	0.35	**< .001**
Sleep T-score	0.18	0.02	**< .001**	0.22	0.04	**< .001**
***PM Males:* n= 86**						
Sleep score	0.88	0.19	**< .001**	1.51	0.32	**< .001**
Sleep T-score	0.23	0.02	**< .001**	0.35	0.04	**< .001**
***NP Males:* n= 78**						
Sleep score	1.09	0.16	**< .001**	1.49	0.26	**< .001**
Sleep T-score	0.18	0.02	**< .001**	0.31	0.03	**< .001**

Note: A Pearson correlation was run on the full preschool cohort (n = 324) to check how well the cross-form determined sleep score aligned with the standard CBCL sleep problems T-scores. The analysis showed a strong, significant positive relationship ($r = .61$, $p < .001$), validating the scoring strategy used in this study.

β: The change in mental health for a 1 unit change in sleep score/sleep T-score
PM: Premutation, NP: No Premutation, SE: Standard Error
Significant *p* -values (*p*) are bold ($p < .05$)
