# Supplementary material for: Parental report–based assessment of sleep problems and mental health in an unreferred cohort of children with a fragile X premutation
Source: Front Mol Neurosci. 2026 Jul 15;19:1820313. doi: 10.3389/fnmol.2026.1820313 (PMC13416670; doi:10.3389/fnmol.2026.1820313)
Supplement: Supplementary file 2 [file Data_Sheet_2.PDF]

Full model results for Table 4: Linear regression analysis of sleep and mental health at Time 1 and Time 2

	T1 Depressive Problems				T1 Anxiety Problems					T2 Depressive Problems				T2 Anxiety Problems			
**All Participants n= 128**	β	SE	*t*	*p*	β	SE	*t*	*p*		β	SE	*t*	*p*	β	SE	*t*	*p*
Intercept	0.68	0.74	0.92	.362	-1.69	1.12	-1.51	.133	Intercept	1.07	1.11	0.96	.338	0.35	1.59	0.22	.828
T1 Sleep Score	1.08	0.15	6.97	**< .001**	1.46	0.23	6.24	**< .001**	T2 Sleep Score	2.01	0.22	9.19	**< .001**	1.75	0.31	5.60	**< .001**
Fragile X status	-0.19	0.24	-0.76	.451	0.79	0.37	2.13	**.035**	Fragile X status	-0.39	0.30	-1.30	.196	0.27	0.43	0.63	.529
Sex	0.16	0.25	0.65	.517	0.30	0.37	0.81	.421	Sex	0.07	0.30	0.24	.808	0.60	0.43	1.41	.16
T1 Age at test	-0.06	0.10	-0.62	.539	0.17	0.15	1.18	.240	T2 Age at test	0.02	0.09	0.17	.863	0.02	0.13	0.18	.855
Race	-0.37	0.32	-1.16	.248	0.11	0.23	0.23	.816	Race	-0.09	0.38	-0.24	.815	0.58	0.54	1.08	.284
Adjusted R^2	0.28				0.25				Adjusted R^2	0.39				0.19			
F-statistic	10.66			**< .001**	9.41			**< .001**	*F*-statistic	17.36			**< .001**	7.01			**< .001**
**PM Females n= 27**																	
Intercept	1.63	1.54	1.07	.298	-0.79	1.95	-0.40	.690	Intercept	-0.47	2.56	-0.19	.855	1.10	2.70	0.41	.687
T1 Sleep Score	1.32	0.42	3.18	**.004**	1.79	0.53	3.40	**.002**	T2 Sleep Score	2.33	0.57	4.11	**< .001**	3.29	0.60	5.50	**< .001**
T1 Age at test	-0.23	0.27	-0.85	.406	0.36	0.35	1.05	.307	T2 Age at test	0.09	0.24	0.38	.704	0.0002	0.25	0.00	.999
Race	-1.17	0.84	-1.40	.174	-0.66	1.06	-0.62	.540	Race	0.03	0.99	0.03	.973	-0.33	1.04	-0.32	.752
Adjusted R^2	0.31				0.25				Adjusted R^2	0.35				0.51			
F-statistic	4.97			**.008**	3.86			**.023**	*F*-statistic	5.75			**.004**	10.13			**< .001**
**NP Females n= 31**																	
Intercept	0.40	1.08	0.37	.714	0.51	1.20	0.42	.676	Intercept	3.71	2.32	1.60	.122	2.72	2.52	1.08	.289
T1 Sleep Score	1.06	0.29	3.61	**.001**	1.37	0.33	4.18	**< .001**	T2 Sleep Score	1.86	0.48	3.88	**< .001**	0.27	0.52	0.53	.603
T1 Age at test	-0.04	0.19	-0.21	.838	-0.06	0.21	-0.28	.780	T2 Age at test	-0.27	0.22	-1.21	.238	-0.04	0.24	-0.16	.874
Race	-1.00	0.56	-1.78	.086	0.30	0.63	0.48	.636	Race	-0.64	0.90	-0.71	.482	0.08	0.98	0.08	.936
Adjusted R^2	0.30				0.41				Adjusted R^2	0.31				-0.10			
F-statistic	5.38			**.005**	7.81			**< .001**	*F*-statistic	5.59			**.004**	0.10			.959
**PM Males n= 35**																	
Intercept	0.98	1.24	0.79	.434	-0.30	2.84	-0.11	.917	Intercept	-0.18	1.26	-0.14	.888	0.04	2.82	0.02	.988
T1 Sleep Score	0.79	0.33	2.37	**.024**	1.84	0.76	2.43	**.021**	T2 Sleep Score	2.08	0.37	5.63	**< .001**	2.30	0.83	2.77	**.009**
T1 Age at test	-0.16	0.21	-0.75	.457	0.25	0.48	0.53	.600	T2 Age at test	0.07	0.11	0.59	.559	0.18	0.26	0.70	.487
Race	0.64	0.66	0.97	.341	-0.24	1.50	-0.16	.876	Race	-0.80	0.58	-1.39	.174	0.61	1.29	0.47	.640
Adjusted R^2	0.20				0.08				Adjusted R^2	0.46				0.15			
F-statistic	3.86			**.019**	2.05			.127	*F*-statistic	10.63			**< .001**	3.03			**.044**
**NP Males n= 35**																	
Intercept	0.15	0.81	0.18	.856	-0.50	1.15	-0.43	..670	Intercept	-1.35	1.75	-0.77	.448	2.24	2.72	0.83	.415
T1 Sleep Score	1.11	0.23	4.71	**< .001**	1.08	0.33	3.23	**.003**	T2 Sleep Score	1.87	0.37	5.04	**< .001**	2.06	0.57	3.60	**.001**
T1 Age at test	0.09	0.14	0.66	.516	0.25	0.20	1.27	.215	T2 Age at test	0.23	0.17	1.35	.188	-0.03	0.27	-0.11	.911
Race	0.14	0.52	0.26	.793	0.71	0.75	0.96	.346	Race	0.80	0.62	1.28	.210	1.16	0.97	1.21	.237
Adjusted R^2	0.38				0.25				Adjusted R^2	0.48				0.28			
F-statistic	7.91			**< .001**	4.70			**.008**	*F*-statistic	11.57			**< .001**	5.32			**.004**

T1 = Time 1 (3–7 years old); T2 = Time 2 (8–13 years old)

β: The change in mental health for a 1 unit change in sleep score

PM: Premutation, NP: No Premutation, SE: Standard Error

Significant *p*- values (*p*) are bold (*p* < *.05)*
